# A study of 1088 consecutive cases of electrolyte abnormalities in oncology phase I trials

**DOI:** 10.1016/j.ejca.2018.08.019

**Published:** 2018-11

**Authors:** Alvaro H. Ingles Garces, Joo Ern Ang, Malaka Ameratunga, Maxime Chénard-Poirier, David Dolling, Nikolaos Diamantis, Satyanarayana Seeramreddi, Raghav Sundar, Johann de Bono, Juanita Lopez, Udai Banerji

**Affiliations:** a The Royal Marsden NHS Foundation Trust, Downs Rd, Sutton, SM2 5PT, London, UK; b The Institute of Cancer Research, 15 Cotswold Road, Sutton, SM2 5NG, London, UK

**Keywords:** Electrolyte abnormalities, Phase I clinical trials, Drug development

## Abstract

**Background:**

The incidence and clinical significance of electrolyte abnormalities (EAs) in phase I clinical trials are unknown. The objective of this study is to evaluate the incidence and severity of EAs, graded according to CTCAE, v4.03, to identify variables associated with EAs and their prognostic significance in a phase I population.

**Methods:**

A retrospective chart review was performed of 1088 cases in 82 phase I clinical trials consecutively treated from 2011 to 2015 at the Drug Development Unit of the Royal Marsden Hospital. Cox regression analysis was performed to examine the relationship between overall survival (OS) and baseline characteristics, treating the occurrence of grade III/IV EAs as a time-varying covariate.

**Results:**

The most common emergent EAs (all grades) were as follows: hyponatraemia 62%, hypokalaemia 40%, hypophosphataemia 32%, hypomagnesaemia 17% and hypocalcaemia 12%. Grade III/IV EAs occurred in 19% of cases. Grade III/IV EAs occurred during the dose-limiting toxicity window in 8.46% of cases. Diarrhoea was associated with hypomagnesaemia at all grades (p < 0.001), hyponatraemia at all grades (p = 0.006) and with G3/G4 hypokalaemia (p = 0.02). Baseline hypoalbuminaemia and hyponatraemia were associated with a higher risk of developing other EAs during the trial in the univariate analysis. Patients who developed grade III/IV EAs during follow-up had an inferior median OS (26 weeks vs 37 weeks, hazard ratio = 1.61; p < 0.001).

**Conclusion:**

This is the first study to demonstrate the clinical significance of baseline hypoalbuminaemia and hyponatraemia, which are predictors of development of other EAs in phase I patients. Grade III/IV EAs are adverse prognostic factors of OS independent of serum albumin levels.

## Highlights

• Patients who develop grade III/IV electrolyte abnormalities have poorer overall survival.
• Hyponatraemia is linked to higher risk of developing other electrolyte abnormalities.
• Hypoalbuminaemia is linked to higher risk of developing electrolyte abnormalities.

## 1 Introduction

The development of molecularly target agents for cancer has resulted in novel adverse events (AEs) correlating with the mechanism of action of these agents. AEs due to anti-cancer treatment are a common form of iatrogenic injury and as molecularly targeted therapies are generally administered continuously, cumulative toxicities can occur [1], [2]. Some AEs caused by this class of drugs are preventable, but many are unanticipated and differ with those of other therapeutics such as conventional cytotoxic agents and immunotherapies. The incidence of metabolic toxicities in phase I studies is not well documented, particularly with regards to electrolyte abnormalities (EAs) and their consequences. These toxicities can range from asymptomatic laboratory findings to symptomatic alterations that can worsen patients' quality of life and lead to death.

The treatment for EAs may range from oral supplementation to anti-cancer therapy interruption and intravenous supplementation, which increases the costs and risks of drug development. Although they appear easier to treat compared with other observable toxicities, the clinical significance of EAs in phase I trials is unknown. In many clinical trials, asymptomatic laboratory toxicities are excluded from dose-limiting toxicity (DLT) assessment as their real clinical significance is doubted. Nevertheless, these toxicities have significant implications on resources, including medical assessment time, laboratory tests, hospital admissions and pharmacy time.

The prognostic significance of some EAs is well described for several tumour types, such as hypercalcaemia for breast and kidney cancer and hyponatraemia for small cell lung cancer [3], [4]. However, the incidence, prevalence and the clinical significance of EAs in oncological phase I studies are not well documented, and the reasons for developing these AEs are poorly understood.

Establishing the prevalence of the electrolyte alterations can help to recognise, prevent and optimally manage them. Furthermore, attempting to understand the risk factors associated with EAs can help refine inclusion/exclusion criteria. To the best of our knowledge, there is no study exploring the overall risk of EAs in the phase I cancer setting. We aimed to study the prevalence of EAs of patients on oncology phase I studies, elucidate potential risk factors and assess their relevance and impact in the drug development process of new agents.

## 2 Materials and methods

The principal objective of this study was to determine the incidence and severity of EAs in a cohort of phase I cancer patients. Secondary objectives were to evaluate the association of EAs with other clinical features and laboratory tests and to estimate the prognostic significance of EAs in the phase I setting. Approval to collect and analyse the data was obtained by applying to the committee for clinical research at The Royal Marsden NHS Foundation Trust as a service evaluation (SE541).

A retrospective chart review was performed of 1088 patient cases with solid tumours in 82 phase I clinical trials consecutively treated from 01/01/2011 to 31/12/2015 in the Drug Development Unit of The Royal Marsden, who were diagnosed with any type of electrolyte disturbance. All data were anonymised before analysis. The clinical and demographics details including age, sex, comorbidities, date of last follow-up and date of death were collected. To be included in this study, patients must have received at least one dose of the experimental drug. The phase I trials included dose escalation and expansion of different classes of drugs, such as protein kinase B (AKT), poly ADP ribose polymerase (PARP), ataxia-telangiectasia and Rad3-related (ATR), Mammalian Target of Rapamycin (mTOR), phosphoinositide-3 kinase (PI3K) and anti-folate receptor inhibitors, used as single agents and/or in combination.

For this project, hypokalaemia, hyperkalaemia, hypocalcaemia, hypercalcaemia, hypomagnesaemia, hypermagnesaemia, hypophosphataemia, hyponatraemia and hypernatraemia were defined and graded according to the Common Terminology Criteria of Adverse Events (CTCAE), version 4.03 [5].

Overall survival (OS) was calculated from date of first treatment to date of death and censored at date of last follow-up. Cox regression was used to examine the relationship between OS and baseline characteristics, treating the occurrence of grade III/IV EAs as a time-varying covariate. Grade III/IV EAs during the first 4 weeks of the trial were analysed using a logistic regression model. Backward stepwise regression with a p-value of 0.2 was used to select variables for a multivariate logistic regression analysis. Impact of different variables such as age, sex, comorbidities and death were analysed. Chi-square and Fisher's exact test were used to evaluate the univariate and multivariate analyses along with 95% confidence interval (CI). All p-values were two-tailed and considered statistically significant if p < 0.05.

## 3 Results

Patient characteristics are listed in Table 1. Fifty-six percent of the patient cases were female, and hypertension was the most common comorbidity (22.4%). Only 5.2% of patients had brain metastases and greater than 92% of patients had normal creatinine. All patients had performance status 0–1.

**Table 1:** Baseline characteristics.

Characteristic	*N*	%
**Gender**		
Male	471	43.3
Female	617	56.7
**Ethnicity**		
White	1017	93.5
Black	13	1.2
Asian	19	1.8
Other	19	1.8
Unknown	20	1.8
**Brain metastases**		
No	1032	94.8
Yes	56	5.2
**Creatinine > ULN**		
No	1044	92.3
Yes	84	7.7
**Comorbidities**		
Hypertension	244	22.4
DM	60	5.5
Hypothyroidism	46	4.2
Hyperthyroidism	7	0.6
DVT/PE	174	16.0

Data related to gender, ethnicity and comorbidities thought to be important in evaluating EAs are described above.

**Fig. 1:**
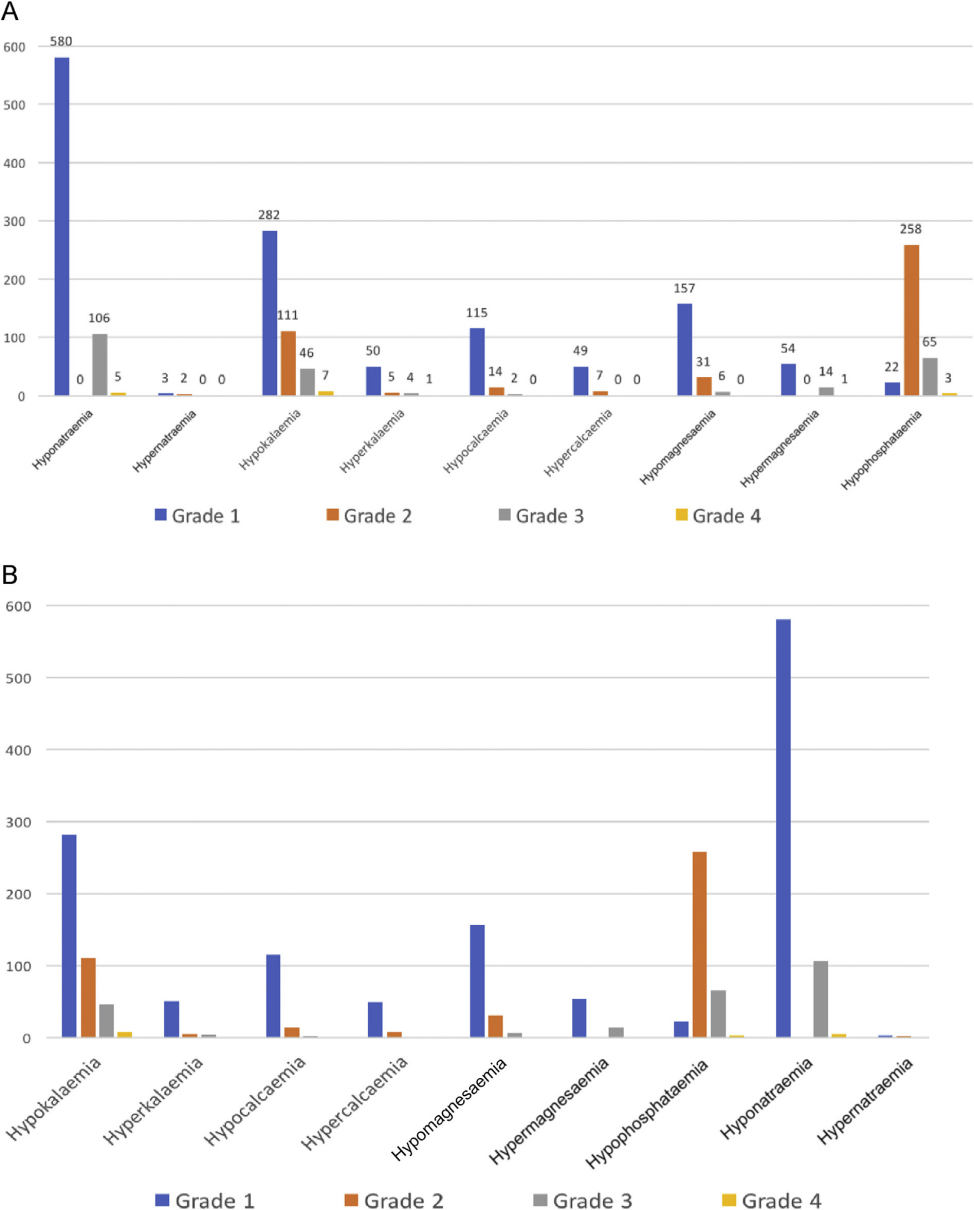
The most common patient cases of electrolyte abnormalities (EAs) in 1088 consecutive patients. (A) The most common emergent EAs recorded during the entire trial or when the data collection stopped (which ever was earlier). (B) The most common emergent EAs during the first 4 weeks of clinical trials, which are typically used to define dose-limiting toxicities.

The most common emergent EAs of all grades during the entire course of trials or the termination of data collection (whichever was first) were as follows: hyponatraemia 62%, hypokalaemia 40%, hypophosphataemia 32%, hypomagnesaemia 17% and hypocalcaemia 12%. Overall, grade III/IV EAs occurred in 19% of cases. More specifically, grade III/IV EAs were observed as follows: hyponatraemia 10%, hypophosphataemia 6%, hypokalaemia 5%, hypomagnesaemia 1% and hypermagnesaemia 1% (Fig. 1A). Importantly, during the first 4 weeks of a phase I trial; typically, the window where the DLT period was assessed, 92 patients (8.46%) had a grade III/IV EA. Fig. 1B shows the incidence of EAs during the first 4 weeks of a phase I trial.

A univariate analysis was done to look for risk factors at baseline (before starting the phase I trial medication) associated with these EAs (Table 2). Baseline creatinine values > upper limit of normality (ULN), baseline values of albumin, sodium and magnesium below ULN were significantly associated with EAs, and these variables remained significant risk factors in multivariate analysis except for magnesium levels below ULN at baseline and baseline creatinine. Importantly, age and comorbidities such as brain metastasis, diarrhoea, hypothyroidism or diabetes were not significant risk factors on either univariate or multivariate analysis. This could have implications on the way in which inclusion/exclusion criteria of phase I studies are established.

**Table 2:** Univariate and multivariate analysis of factors affecting EAs.

	Univariate			Multivariate		
	OR	95% CI	*p*-value	OR	95% CI	*p-*value
Age (per 10 years)	1.19	0.99–1.42	0.06	1.14	0.94–1.37	0.19
Gender						
Male	1	–	0.4	–	–	–
Female	1.21	0.78–1.87	–	–	–	–
Ethnicity						
White	1	–	0.27	–	–	–
Black	0.94	0.12–7.30	–	–	–	–
Asian	3	0.97–9.25	–	–	–	–
Other	2.11	0.60–7.39	–	–	–	–
Unknown	0.59	0.08–4.48	–	–	–	–
Diarrhoea	0.94	0.59–1.49	0.78			
Vomiting	1.46	0.95–2.24	0.08	1.52	0.97–2.38	0.07
Brain Mets	0.83	0.29–2.34	0.72			
Creatinine > ULN	2.37	1.28–4.41	**0.006**	2.73	1.42–5.26	**0.003**
Comorbidities						
Hypertension	1.41	0.87–2.27	0.16	–	–	–
DM	1.22	0.51–2.91	0.66	–	–	–
Hypothyroidism	1.03	0.36–2.95	0.95	–	–	–
DVT	0.94	0.52–1.70	0.83	–	–	–
Hypercholesterolaemia	1.13	0.47–2.69	0.79	–	–	–
Osteoporosis	2.44	0.52–11.45	0.26	–	–	–
Coronary disease	0.45	0.06–3.33	0.43	–	–	–
**Baseline lab results**						
Creatinine (per 10)	1.06	0.95–1.17	0.3	1.11	0.98–1.24	0.06
Albumin (per 10)	0.32	0.20–0.51	<**0.001**	0.53	0.32–0.86	**0.01**
Na	0.74	0.69–0.80	<**0.001**	0.76	0.70–0.82	<**0.001**
K	0.73	0.41–1.30	0.28	–	–	–
Ca	1.88	0.59–5.98	0.29	–	–	–
P	0.69	0.30–1.58	0.38	–	–	–
Mg	0.76	0.62–0.93	**0.008**	–	–	–

The figures in bold represent statistically significant in both univariate and multivariate analysis.

We also studied associations of individual EAs to other concomitant toxicities. It was found that diarrhoea was associated with hypomagnesaemia in all grades (hazard ratio [HR] 1.78, 1.32–2.39 95% CI, p < 0.001), with grade III/IV hypokalaemia (HR 1.93, 1.09–3.43 95% CI, p = 0.02) and with hyponatraemia in all grades (HR 0.79, 0.67–0.93, 95% CI, p = 0.006) as well. Vomiting was also associated with hypomagnesaemia in all grades (HR 1.45, 1.08–1.95 95% CI, p = 0.01) and grade III/IV hypokalaemia (HR 2.91, 1.62–5.23, 95% CI, p < 0.001). Baseline hypoalbuminaemia (odds ratio [OR] 0.32, 95% CI 0.20–0.51, p < 0.001) and hyponatraemia (OR 0.74, 95% CI 0.69–0.80, p < 0.001) are associated with higher risk of developing other EAs on trial in the univariate analysis.

**Fig. 2:**
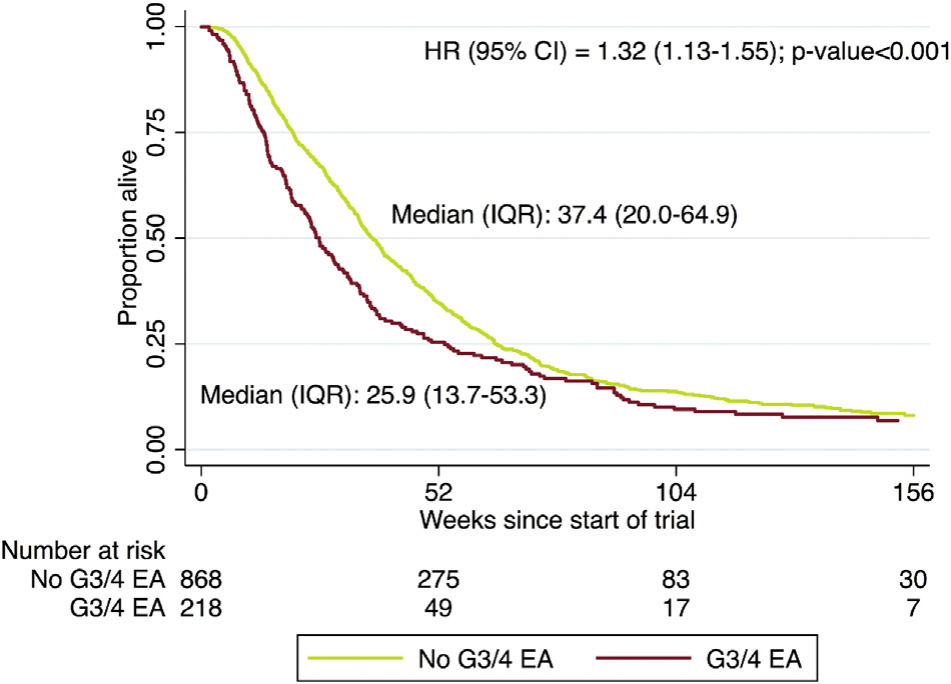
Overall survival of patients with and without grade III/IV EAs. A Kaplan–Meier estimate of survival of patients who did and did not experience a grade III/IV EA during the entire period of the trial or when data collection was stopped (whichever was earlier). HR, hazard ratio; CI, confidence interval; IQR, interquartile range; EA, electrolyte abnormality.

Patients who developed grade III/IV EAs during the period of the phase I study had a poorer median OS (26 weeks vs 37 weeks, HR = 1.61; 95% CI: 1.37–1.90; p < 0.001) (Fig. 2).

## 4 Discussion

Baseline EAs are common in patients with advanced cancer participating in phase I trials. However, the incidence, prevalence and clinical prognostic significance of EAs in phase I studies are not well documented. To date and to our knowledge, this is the first detailed evaluation of electrolyte panel alterations and its implications in cancer care in the phase I setting, providing data of special relevance to the drug development process.

Improvement in cancer outcomes has been observed over the last few decades; however, it has unveiled newer challenges including different metabolic abnormalities. It is reported that hypophosphataemia is a frequent adverse effect of mTOR inhibitors, MET and selective ALK inhibitors [6], [7]. mTOR inhibitors could downregulate phosphate carriers in the proximal tubules along with increased 1,25-dihydroxyvitamin D3 levels in a preclinical study [6]. Hypomagnesaemia is a common metabolic abnormality in treatment with monoclonal antibodies against endothelial growth factor receptor (EGFR) [6]. A prospective analysis showed defective renal magnesium reabsorption, which is thought to arise from the role of EGFR in regulating the activity and distribution of transepithelial magnesium TRPM6 [6], [8]. Despite all this information, we do not have much data about these EAs with other agents and disturbances of sodium, potassium and calcium with the wide range of anti-cancer target therapies that we have today. The reasons for developing these side-effects are poorly understood and many pathophysiologic mechanisms have been proposed. The relevance of our study is that it shows that EAs in general are not only a theoretical risk but also a real and pragmatic issue.

Early diagnosis of EAs and appropriate management are important and expected to reduce adverse outcomes. However, the experience of a significant toxic event in a patient with a poor prognosis has clinical and quality of life implications. The clinical presentation of EAs is variable, ranging from asymptomatic to minor manifestations such as fatigue, to more serious and life-threatening manifestations such as cardiac arrhythmia. Clinicians treating patients in phase I trials should be able to define the risk associated with experimental treatments to assist patients in undertaking the decision to undergo such therapies as patients often underestimate the impact of significant treatment-related toxicity associated with phase I agents [1].

Although diagnosis of EAs is relatively simple with routine laboratory assessments on biochemical panels, identifying the causal mechanism of EAs is more problematic. Possible causes in cancer patients include the use of a large number of concomitant medications, some of which are known to cause EAs [9], [10], as well as cancer-induced organ dysfunction such as renal impairment [11], which can also commonly cause EAs. Malignancy itself is also associated with paraneoplastic phenomena that manifest as EAs. Well-known cancer-associated metabolic disturbances include hypercalcaemia of malignancy and hyponatraemia induced by syndrome of inappropriate ADH secretion [12], [13], [14].

Hyponatraemia is known to be the most common EA in clinical practice. It is associated with poor clinical outcomes such as reduced survival, disability, prolonged hospital stay and increased hospital costs [12], [13], [14], [15], [16]. Published data suggest that hyponatraemia, even when mild and chronic, represents an economic burden [16]. Therefore, it is not surprising that hyponatraemia is associated with an increased resource utilisation and costs [16]. This is an important issue that needs to be considered as the costs of drug development could be increased if hyponatraemia and other EAs are underestimated. To exemplify this condition, in the United States of America, the direct medical costs of hyponatraemia in a general population were estimated to range between $1.6 billion to $3.6 billion [16], [17]. Despite these data, hyponatraemia is often poorly considered if not ignored, even in a cancer population, as well as other EAs which we have no estimation of their social burden.

As reported previously, electrolytic disorders are common in cancer patients and may worsen patient prognosis. Hyponatraemia in small-cell lung is well correlated with prognosis and survival [12], [15], [18]. Few studies have specifically focused on non-small-cell lung cancer patients, but it has been shown that the normalisation of sodium concentration improved OS and progression-free survival (PFS) in this population [19]. However, our study is the first one to demonstrate the clinical significance of baseline hyponatraemia with development of other EAs and that grade III/IV EAs are significant adverse prognostic factors of OS in phase I patients with different tumour types. Thus, sodium is a very important parameter that could be added to validated prognostic scores used to select patient for clinical trials.

In the multivariate analysis, comorbidities such as hypertension, diabetes or hypothyroidism were not significantly associated with EAs during phase I trials. It is important to highlight that, contrary to expectations [20], in the analysis of our data, presence of brain metastases had no clinical significant association with EAs, including hyponatraemia. This is probably because few patients with brain metastases were enrolled on trials as this condition is usually an exclusion criterion for phase I studies.

In phase I trials, a strong association between the EAs hypokalaemia/hypomagnesaemia and the AEs vomiting/diarrhoea was demonstrated. Although this association is well known regardless of the context [21], [22], our study shows that those EAs may be better objective measures of drug-related toxicity than the current CTCAE criteria for patient-reported diarrhoea/vomiting, as they are highly subjective and open to recall bias. This could have direct clinical implications when dealing with drugs known to cause these AEs, and hypokalaemia/hypomagnesaemia could be used as surrogate markers of gastrointestinal toxicities.

Our descriptive epidemiological study has some important advantages in its design: using data from patients enrolled into phase I clinical trials, it was reassured that the studied population would not have major organ dysfunction as baseline and high-quality data without missing values were available leading to reliable results. Another strength of this study is the large sample size; therefore, EAs could be investigated and conclusions could be drawn accordingly. However, despite the large size of this cohort, some limitations need to be considered. This is a retrospective study with a heterogeneous cohort not only in terms of tumour types but also the class of drugs and their combinations used in different phase I trials. Moreover, the studies were conducted in a specialised phase I cancer centre, so it does not reflect the general patient population but, on the other hand, the numbers are robust enough to allow conclusions in this very specific population. The patient cases collected were on 82 different clinical trials. While it would have been interesting to compare EAs between different drugs or different classes of drugs, getting permission to do so from sponsors in all cases was thought to be impractical and not feasible.

Currently, most phase I studies do not have cutoffs for EAs in their exclusion criteria, but their focus is on haematological, renal and liver function tests. Our data suggest that abnormal baseline EAs not only predict AEs related to EAs but also prognosis and should be considered while establishing inclusion and exclusion criteria. Similarly, baseline albumin has been recognised as predictor for survival and AEs [23].

There is no specific test that will establish the cause of drug-induced metabolic alterations, but if any EA is recognised, differential diagnosis and the liaison between target-therapy and electrolyte alteration could be made. Establishing the diagnosis of drug toxicity is important, as it may have significant implications for clinical care, as measures for prevention and correct management will be taken and the discontinuation of an agent on suspicion alone could be avoided and the patient would not be deprived of a potentially life-prolonging treatment. EAs can be another tool to help how to improve patient selection for clinical trials and to reduce the likelihood of expensive failures during the drug development process. Given the risk and the high incidence of EAs observed in this study, careful monitoring and early treatment are proposed as EAs can worsen the performance status and patient's quality of life. These results can improve the safety of phase 1 clinical trials and also it will be a useful tool for future reference in medical research as a definitive study of EAs in phase I clinical trials setting.

## Conflict of interest statement

All authors declare no known conflicts of interest for this manuscript.
